# Experimental dissection of tuberculosis protective immunity: a human perspective

**DOI:** 10.3389/fcimb.2025.1595076

**Published:** 2025-06-30

**Authors:** Sarah Schmidiger, Damien Portevin

**Affiliations:** ^1^ Department of Medical Parasitology & Infection Biology, Swiss Tropical and Public Health Institute, Allschwil, Switzerland; ^2^ Faculty of Science, University of Basel, Basel, Switzerland

**Keywords:** tuberculosis, immunology & infectious diseases, human, model, systems immunology

## Abstract

*Mycobacterium tuberculosis* (*Mtb*), the causative agent of tuberculosis (TB), has plagued humankind for millennia. Claiming 1.25 million lives in 2023, TB remains the worldwide leading cause of death from a single-infectious agent. Improved vaccines, diagnostics and treatment regimens for drug-susceptible and drug-resistant cases are paramount to attain the goals of the WHO’s End TB Strategy. Our knowledge gap in protective immunity in TB impedes the development of such new vaccines and host-directed interventions. *Mtb* is a pathogen highly adapted to humans and primarily infects the lungs. Access to relevant specimens is invasive, preventing ample human TB studies, which therefore mostly rely on peripheral blood specimens and biopsies. Thus, there is a need for relevant surrogates. In recent years, *in vivo*, *in vitro*, and *in silico* systems have arisen to approach and model different aspects of TB pathogenesis. Moving away from cell-line infections and classical animal models, TB research has advanced to genetically diverse mice, 3D organoid cultures and computational modelling. We will review current TB models and discuss their applicability to decipher protective human immunity, understand disease progression, transmission, as well as evaluate vaccine candidates and unravel host-directed therapeutic approaches.

## Introduction

Tuberculosis (TB) elimination remains an ambitious target. Despite extensive research, this ancient disease keeps claiming 143 lives every hour, making *Mycobacterium tuberculosis* (*Mtb*) the leading single-infectious killer (WHO, 2024). The WHO has outlined a strategy to end the global TB epidemic by 2035. This includes reducing mortality by 95% and incidence by 90% compared to 2015. To reach those goals, we urgently need new vaccines, improved drug regimens and innovative, host-directed interventions. Owing to ethical restrictions as well as limited access to *in situ* samples, human TB studies are scarce. Thus, there is a need for appropriate surrogates that allow us to decipher protective traits against this pathogen, which after millennia of co-evolution is highly adapted to humans. Intriguingly, while a substantial proportion of the human population is thought to be latently infected (estimates ranging between 25 and <10% (Houben and Dodd, 2016; Schwalb et al., 2024)), only <5% of encounters with *Mtb* result in active TB disease (Behr et al., 2018). Progression to active disease is dependent on the strain, the age and the intensity of exposure (Sloot et al., 2014; De Jong et al., 2008). In contrast, most current infection systems fail to capture the 90-95% of protective outcomes. Hence, several susceptibility traits to TB, such as impaired T cell function or IL-12/IFN-γ and TNF-α signaling, have successfully been dissected by combining research in animals, humans and *in vitro* systems with epidemiological data (Scriba et al., 2017; Bustamante et al., 2014; Wallis, 2007). We have further come to realize that environmental factors (e.g. nutrition, economic status) may be as influential as genetic and immunological ones (Bhargava et al., 2023). Historically, studying resistance to disease over susceptibility has greatly contributed to improving public health by delivering the smallpox vaccine (developed after observing cowpox-exposed milkmaids resisted the disease) and CCR5-inhibitors for HIV treatment (after realizing that individuals carrying a CCR5 variant are HIV-resistant). A similar approach could be useful for TB too, for our understanding of TB protective traits is very limited. With ongoing efforts to recruit and characterize natural or vaccine-induced “resister” cohorts, research into TB resistance is gaining momentum. We here review recent *in vivo*, *in vitro* and *in silico* approaches that may capture and dissect protective traits in TB, which in turn could be leveraged for vaccine and host-directed therapy (HDT) drug design. Particularly, we emphasize the potential of human-based *in vitro* approaches, combined with advanced technologies and computational modeling, as promising tools to characterize protective immune mechanisms and to serve as clinical platforms for vaccine and drug development.

## *In vivo* studies

### *In vivo* studies in animals

The first experimental infections of guinea pigs with *Mtb* date back to 1882, when Robert Koch identified *Mtb* as the causative agent of TB. Since, animal models ranging from amoeba and zebra fish, over rodents to cattle and non-human primates have been invaluable to increase our understanding of TB pathogenesis. Animal models for TB have been extensively reviewed elsewhere (Ernst, 2012; Dube et al., 2020; Singh and Gupta, 2018; Williams and Orme, 2016; Bucsan et al., 2019) and more specifically in light of vaccine (Gong et al., 2020) and chemotherapy (Yang et al., 2021) development. Here, we will emphasize limitations and advantages of selected animal models to study protective immunity in human TB and discuss ongoing efforts in overcoming their caveats.

**Figure 1:**
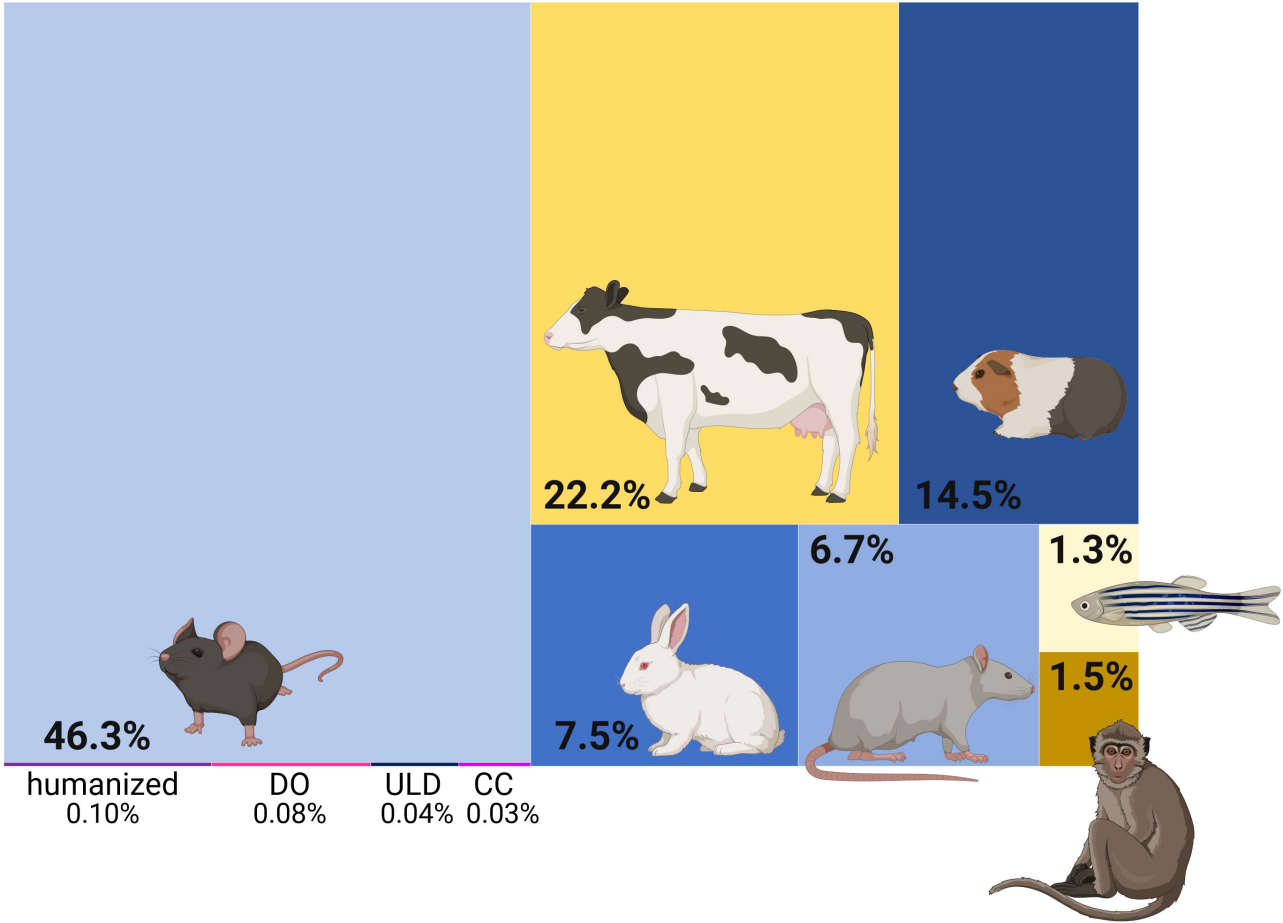
Tuberculosis *in vivo* studies in animals. Treemap representing the proportion of publications using the respective animal models. Yellow shades indicate challenge models of natural host-pathogen pairs. DO diversity outbred; ULD ultra-low dose; CC collaborative cross. Generated with biorender.com; Based on a PubMed search on 21.11.24 using the queries: “tuberculosis” AND (“mice” OR “mouse”) | (“mice” OR “mouse”) AND (“ULD” OR “ultra-low dose”) | (“mice” OR “mouse”) AND (“collaborative cross”) | (“mice” OR “mouse”) AND (“diversity outbred” OR “DO”) | (“mice” OR “mouse”) AND (“humanized mice”) | “cattle” | (“guinea pigs” OR “guinea pig”) | (“rabbits” OR “rabbit”) | (“rats” OR “rat”) | (“zebrafish” OR “zebra fish”) | (“NHPs” OR “NHP” OR “non-human primate*” OR “nonhuman primate*”).

*Mtb* is one of several TB-causing pathogens, collectively known as the *Mycobacterium tuberculosis* complex (MTBC). The MTBC encompasses 10 lineages infecting humans, with lineages 1 to 4 accounting for 99% of TB cases, and nine animal infecting ones (Goig et al., 2025). Zoonotic TB can occur in humans, e.g. infection with the bovine pathogen *Mycobacterium bovis* following ingestions of unpasteurized milk (Olea-Popelka et al., 2017), which adds to the global TB burden in areas of human contact with live-stock. Rare instances of TB reactivation from latent *M. bovis* infection have been reported (Capoferri et al., 2024). While TB also exists in animals, MTBC strains that infect humans are highly restricted to their host and failed to induce lung lesions in cattle (Villarreal-Ramos et al., 2018). *Mtb* has plagued and coevolved with humans for thousands of years (Gagneux, 2012). In that regard, experiments conducted in animals, aiming to decipher TB in humans, can legitimately be criticized to “pervade the field” (Scriba et al., 2017). Due to the global spread of institutional facilities and availability of an arsenal of genetic and immunological tools, mice constitute the most commonly used model in TB *in vivo* studies (**Figure 1**). This is yet very concerning, as mice are no natural host for *Mtb* and are not naturally infected by any MTBC member. Nonetheless and as elegantly reviewed elsewhere (Ernst, 2012), animals can serve as surrogates of specific stages in the “immunological life cycle” of tuberculosis. Guinea pigs and mice model the delayed onset of adaptive immunity, rabbits capture necrosis and lung cavitation, NHPs and mice reproduce the impact of CD4 T cell defects or NHPs and cattle allow to study latency and reactivation. Mouse breeds used in TB research include susceptible (C3HeB/FeJ, DBA/2, 129/Sv) and resistant (BALB/c and C57BL/6) mice (Soldevilla et al., 2022). However, a common denominator across mouse models is that they cannot eliminate the infection and do not reflect the large majority (>95%) of human infection outcomes. Efforts to develop mouse models that may better reflect human TB pathogenesis are being pursued (Yang et al., 2021). In 2021, Kevin Urdahl’s group reported an ultra-low dose (ULD) aerosol challenge model (Plumlee et al., 2021). Unlike other infection models, ULD-infected mice responded heterogeneously to infection and remarkably showed singular, organized granulomas. Furthermore, the authors were able to extract blood transcriptional signatures that correlated with disease severity in NHPs and predict TB risk in humans. Aside from optimized infection doses, advances are being made to account for genetic diversity. Collaborative cross (CC) or diversity outbred (DO) mice are deployed to address how the genetic background may influence immune responses and vaccine efficacy (Saul et al., 2019). The use of humanized mice, in which human immune cells are engrafted into immunodeficient mice, is also emerging and their applications recently reviewed (Mcdonald et al., 2024). Humanized mice have been utilized in TB research as promising tools in the assessment of drugs (Arrey et al., 2019), bacteriophage therapy (Yang et al., 2024), innovative vaccines strategies (Afkhami et al., 2023), and to dissect HIV-*Mtb* co-infection (Bohórquez et al., 2024; Calderon et al., 2013). Together, these advanced murine models hold promise in overcoming some of the caveats of standard mouse research for TB, e.g. more diverse infection outcomes, while still benefitting from genetic manipulation avenues. ULD, CC and DO mice may provide valuable data for vaccine development (Wang et al., 2024) and new insights into protective or detrimental immune traits. However, their use – together making up a mere 0.25% of publications - is still in its infancy (**Figure 1**).

Despite its importance for human interventions, the transmission feature of human TB disease is understudied (Behr et al., 2020). Nevertheless, guinea pigs are particularly susceptible to *Mtb* infection and like humans; they can transmit the disease via aerosols (Lurie, 1930). An intriguing study revealed that a glycolipid component of the *Mtb* cell wall (sulfolipid-1) is able to induce cough in guinea pigs (Ruhl et al., 2020). Rabbits constitute important models in TB research, which has been reviewed elsewhere (Bucsan et al., 2019). Noteworthy, the rabbit was the first animal model able to mimic HIV-immune reconstitution inflammatory syndrome (IRIS) using corticosteroids (Manabe et al., 2008). Besides, rabbit infection with *Mtb* notably allowed studying the dynamics of cavitary disease and found a significant degree of molecular and pathological correlates with humans (Kübler et al., 2015). Studies in zebra fish (infected with *M. marinum*) and cattle (infected with *M. bovis*) provide an interesting opportunity to study TB in the context of natural host-pathogen pairs; the former being much easier to use and the latter genetically closer to humans. Interestingly, the zebra fish model has provided mechanistic insights into human bone TB. In an elegant study ignited by a TB outbreak with atypical rates of disseminated and skeletal TB, Saelens et al. linked occurrence of bone TB to increased macrophage motility, dependent on the presence of *esxM* (Saelens et al., 2022). This gene is present in its full length in ancestral lineages, while modern lineages carry a truncated form. This discrepancy, in conjunction with host factors (Rachwal et al., 2024), supports the association of particular ancient lineages with extra-pulmonary TB (EPTB) (Click et al., 2012; Du et al., 2023). Models of natural host-pathogen pairs also allow assessing the conservation of protective or susceptibility traits across different species. This is particularly relevant as zoonotic TB associated to *M. bovis* infections can cause human deaths (Capoferri et al., 2024) and *M. marinum* can cause skin infections in humans (Gonçalves et al., 2022). Non-human primates (comprising cynomolgus monkeys, rhesus monkeys and marmosets (Yang et al., 2021)) display TB pathology and disease spectrum closely resembling those of humans (Scanga and Flynn, 2014), likely rendering them the most relevant model of human TB. Cynomolgus macaques (Capuano et al., 2003) are particularly suitable to study latent infection (O'Garra et al., 2013). However, financial, logistical and ethical concerns imped the wide adoption of these models. Nonetheless, NHPs have proven highly valuable in highlighting human-like heterogeneity of disease presentation across animals and across individual granulomas within a single animal (Lin et al., 2014). Recently, NHP studies yielded further insights into correlates of protection associated with the presence of a NK cell subset in the lungs of latently infected macaques (Esaulova et al., 2021). Comparison of low and high-burden granulomas in cynomolgus macaques also suggested correlates of bacterial clearance associated to the accumulation of T_H_17 and cytotoxic T cells (Gideon et al., 2022). Interestingly, a parallel study also identified cytotoxic signatures to be associated with protection (Winchell et al., 2023). Hansen et al. made a breakthrough by demonstrating unprecedented sterilizing immunity following vaccination with a cytomegalovirus-vectored TB vaccine (RhCMV/TB) (Hansen et al., 2018). Later, similar successes were achieved using an intra-venous (IV) BCG vaccination route (Darrah et al., 2020; Larson et al., 2023). IV BCG vaccination induced protective humoral responses (IgM titers, complement) and NK cell activation in a dose-dependent manner (Irvine et al., 2024). In a separate study, the recruitment and priming of alveolar macrophages and polyfunctional T cells characterized the lung response of protected animals (Peters et al., 2025). Another study demonstrated the necessity of CD4 T and innate CD8 lymphocytes, but not adaptive CD8 lymphocytes, for IV BCG-mediated protection (Simonson et al., 2025). Altogether, recent data collected through the IV BCG approach in NHPs suggest that a plethora of immune players is likely required to synergize for protection.

Overall, animal models have proven valuable to dissect different aspects of TB pathogenesis (Soldevilla et al., 2022). However, controversy remains in the extent of translatability of these findings across different models and, most importantly, to humans (Warren et al., 2015). One of few studies directly comparing immune responses to *in vivo Mtb* infection across DO mice, NHPs and humans found a great degree of overlap of genes differentially expressed during TB disease (Ahmed et al., 2020). Yet, animal models are raising controversial conclusions. For example, a study in mice found a therapeutic potential of MAIT T cells, where their expansion induced by an antigen increased bacterial control (Sakai et al., 2021a); however, in NHPs, the same treatment induced exhaustion of MAIT T cells (Sakai et al., 2021b). *In vitro* models (detailed in the sections below), have highlighted further similarities and discrepancies. Corroborating Ahmed et al., a recent preprint found human and murine alveolar macrophage responses to *Mtb* infection to be majorly conserved; however, they also identified several pathways (e.g. cholesterol, IFN genes) that differ between the two species (Dill-Mcfarland et al., 2025). Other studies have highlighted that rapamycin-induced autophagy restricts *Mtb* replication in murine (Gutierrez et al., 2004), while it would promote it in human macrophages (Andersson et al., 2016). This lack of total translatability is inevitable, given the inherent, irrevocable differences between animals and humans, and the respective TB pathogeneses. While advanced animal models retain relevance as *in vivo* surrogates and will remain an essential tool in pre-clinical safety assessment, scientific efforts are being made to focus translational TB research on human-based approaches. In that perspective, we will now review available approaches aiming at identifying protective traits in humans or human-based systems.

### Human *in vivo* studies

Studying TB in humans is challenged by legitimate ethical considerations and the particularly invasive access to the primary infection sites (lung, lymph nodes, bones or brain), making diseased and non-diseased specimens globally scarce. Consequently, most *in situ* specimens are derived from biopsies or lung/lymph node resections that mostly reflect late stages of failed immune responses and do not allow studying the early onset of disease nor protective traits. Nonetheless, such specimens have provided valuable insights into the spatial organization (Marakalala et al., 2016) of late-stage human TB granulomas, as well as unexpected immunoregulatory features (McCaffrey et al., 2022).

To understand protective immunity, it appears paramount to study infection and ensuing pathogenesis from its earliest onset. Such investigations have become possible through controlled human infection model (CHIM) studies (Pollard et al., 2012) that have already yielded exciting results in vaccine efficacy and drug assessment for the Malaria field (Sauerwein et al., 2011). In TB, the first CHIM trial was conducted over a decade ago, first with intra-dermal (Minassian et al., 2012) and later aerosol-administration of a live-attenuated BCG vaccine strain (Satti et al., 2024). Currently, conditionally replicating *Mtb* strains are being explored as a BCG replacement (Wang et al., 2024; Balasingam et al., 2024). Specifically, a triple-kill-switch *Mtb* strain may present a safe and more physiological candidate for CHIM studies (Wang et al., 2025), holding promise to propel TB vaccine development. While mainly used to assess vaccine efficacy, insights into protective immunity are expected to arise from samples collected alongside CHIM studies. Similarly, a growing body of investigations benefit from existing vaccination cohorts to seek biomarkers of (i) correlates of protection, by identifying vaccine-induced markers that are respectively absent and present in individuals that later progressed or not to active disease; (ii) correlates of risk, with markers whose presence/absence are respectively associated with a low or a high risk of disease, reviewed in (Scriba et al., 2017; Walzl et al., 2011). Yet, vaccination cohorts do not allow identification of naturally occurring protective traits that do not necessarily overlap with vaccine-induced correlates of protection. In that context, the identification of “TB resisters” sparked new hope in the identification of protective traits in human TB. Resisters are defined as individuals repeatedly exposed to *Mtb* that do not harbor detectable immune-reactivity to mycobacterial antigens by TST and/or IGRA testing (Simmons et al., 2018). Study of resister phenotypes is hampered by limitations in the classification of resisters, linked to the lack of tools to assess and estimate the degree of exposure, which is under the influence of the index case’s TB disease status, including bacillary load, cough and strain diversity affecting transmission rate (Simmons et al., 2018). Yet, TB resistance in the absence of adaptive immune memory supports an exceptional potency of innate myeloid responses, which have been shown to be epigenetically regulated in a cohort of health care workers (Zhang et al., 2021). Evidence from historical and contemporary studies suggest that the frequency of resisters is below 10%, although high inter-study variability is observed (Simmons et al., 2018). Recent data suggest that this figure may be over-estimated due to IFN-γ independent and regulatory responses, such as those characterizing the T cell response of household contacts in Kampala (Sun et al., 2024). Further research is necessary to untangle the importance of the various mechanisms supporting natural resistance to *Mtb* infection; and potentially exploit them in host-directed therapeutic approaches. Such approaches may encompass training innate immune responses, and/or boosting IFN-γ-independent cellular mechanisms mediated by lipid-specific MAIT or γδ T cells at the instar of peptide-specific T cells measured in IGRAs. Furthermore, B cells and antibodies might play an additive role (Simmons et al., 2018). Investigation of these resister phenotypes constitutes exquisite translational research for which data from human-based *in vitro* systems may nicely complement animal challenge models. Specimens of resister cohorts may indeed be subjected to an arsenal of *in vitro* tools (described in the section below) to extract features and demonstrate mechanisms of TB resistance.

**Figure 2:**
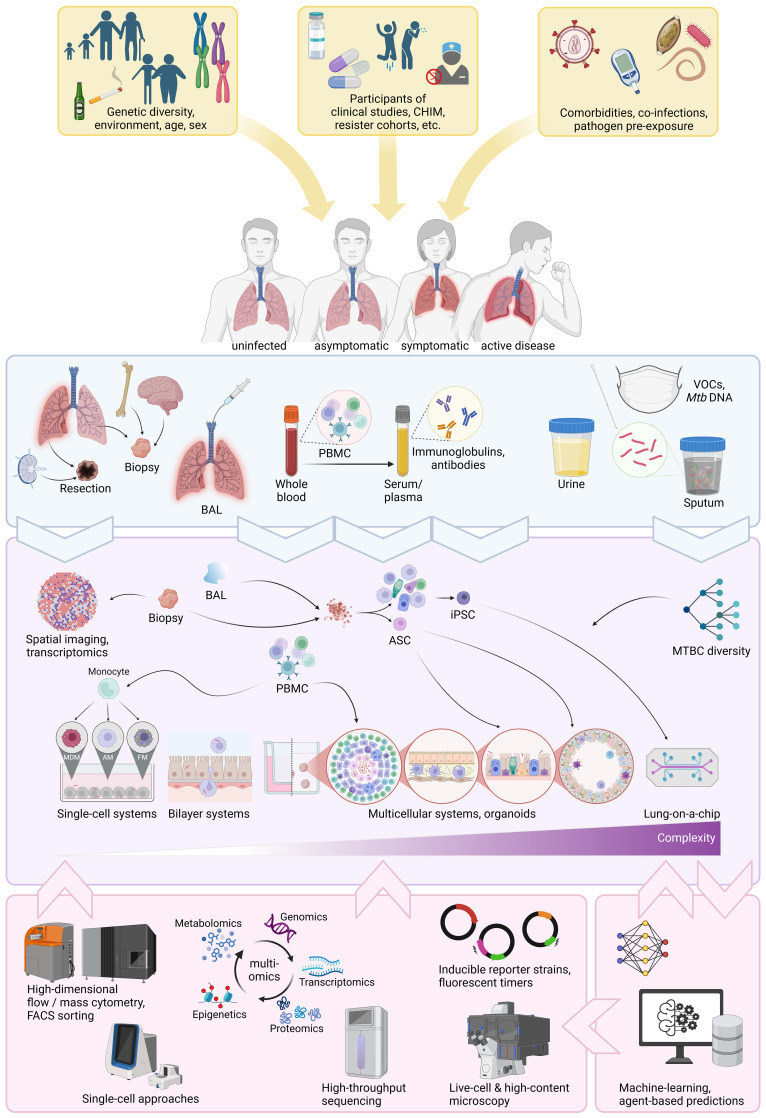
Overview of current human-based approaches to decipher protective immunity. Sources of human diversity, co-factors, co-morbidities, co-infections and disease spectrum (yellow boxes and arrows). Sources of human specimens (light blue box). Human specimens obtainable from those sources and *in vitro* approaches human specimens can be used for (purple box). Cutting-edge technologies and computational approaches that can be applied to *in vitro* systems (pink boxes). CHIM, controlled human infection model; BAL, broncho-alveolar lavage fluid; PBMC, peripheral blood mononuclear cell; VOCs, volatile organic compounds; ASC, adult stem cell; iPCS, inducible pluripotent stem cell; MDM, monocyte-derived macrophage; AM, alveolar macrophages; FM, foamy macrophage; FACS, fluorescence-activated cell sorting. Generated with biorender.com.

In summary, human *in vivo* studies encompassing vaccine and drug trials, resister cohorts and controlled human infections are generating a multitude of specimens that can now be used in human-based *in vitro* approaches to further dissect protective traits (**Figure 2**). This is of particular relevance, since human trials are lengthy and costly. It is thus paramount to make informed choices on bio-banking relevant specimens to pursue the most promising leads. We will now seek to outline the newest advances in that respect.

## Human *ex vivo* and *in vitro* studies

This section highlights human-derived TB *in vitro* studies, summarized in **Table 1**. *In vitro* infections of macrophages to study TB date back to the 1940s (Lurie, 1942). Since then, technological advances surged from alveolar and foamy macrophages, over bilayer systems and 3D granuloma models all the way to lung tissues and stem cell-derived organoids, even extending to entire organs (**Figure 2**). *In vitro* approaches based on human specimens hold great promise to assess comorbidities and incorporate the complex dimension of human genetic diversity. Some are scalable and might find applications for drug screening.

**Table 1:** Overview of human *in vitro* TB infection systems.

Model (ascending complexity)	Technical difficulty	Cell source	Can be donor-specific	Applications*	Reference(s)
THP-1	–	Cell line	NO	H-P interaction; drug-screening (HTS)	Shah et al., 2022
MDM	+	PBMC	YES	H-P interaction; drug-screening (HTS)	Vogt and Nathan, 2011
AM	++	PBMC	YES	H-P interaction; innate alveolar response	Pahari et al., 2024
FM	++	PBMC	YES	H-P interaction; *Mtb* persistence	Daniel et al., 2011; *reviewed by* Santucci et al., 2016
iPSC	++	PBMC, BAL	YES	H-P interaction; role of specific genes; drug-screening (HTS)	Aylan et al., 2023
Alveolar barrier	++	Cell line, PBMC	(YES)	Innate alveolar responses	Birkness et al., 1999; Bermudez et al., 2002
2D granuloma models	+	PBMC	YES	Innate and adaptive responses; drug-screening (HTS); HDT	Puissegur et al., 2004; Guirado et al., 2015
3D granuloma models	++(+)	PBMC	YES	Innate and adaptive responses; *Mtb* resuscitation; HDT	Kapoor et al., 2013; Tezera et al., 2017; Arbués et al., 2020b; *reviewed by* Elkington et al., 2019
Spheroid granulomas	+++	BAL, PBMC	YES	Innate and adaptive responses; HDT	Kotze et al., 2021
Miniaturized TB spheroids	+++	THP-1/PBMC, cell line fibroblasts	YES	Innate and adaptive responses; HDT	Mukundan et al., 2021a
ALI-PBEC	++++	Human lung tissue	YES	Innate alveolar responses; HDT	Barclay et al., 2023
Experimental human lung tissue	++++	Cell line fibroblasts, epithelial cells, PBMC	(YES)	Innate alveolar responses; HIV co-infection; drug-screening; HDT	Hoang et al., 2012 *model*; Parasa et al., 2014 *Mtb application*
Human bronchial airway organoid model	+++++	Human lung tissue (ASC)	YES	Innate alveolar responses, HDT	Iakobachvili et al., 2022
Human lung organoid	+++++	Human pluripotent stem cells (H9 cell line)	(YES)*	Innate alveolar responses; drug-screening; HDT	Kim et al., 2024
Lung-on-a-chip	★★★★★	Primary murine lung epithelial and endothelial cells, BM	(YES)*	Innate alveolar responses; drug-screening; HDT	Huh et al., 2010 *model*; Thacker et al., 2020 *Mtb application*

*can be combined with MTBC diversity.

ALI-PBEC, air-liquid interface model of human primary bronchial epithelial cells; ASC, adult stem cells; HTS, high-throughput screening; HDT, host-directed therapy; PBMC, peripheral blood mononuclear cells; H-P, host-pathogen. (YES) partially, because cell lines involved; (YES)* theoretically feasible but not yet done for *Mtb.*Symbols represent technical difficulty from very easy (-) to highly demanding (+++++) and extraordinarily demanding (stars).

### Single-cell systems

*Mtb* can survive and thrive in the very cells deployed for its clearance. Consequently, macrophages have long been the center of attention in TB research. Numerous infection systems exist to study the host-pathogen interaction between *Mtb* and macrophage host cells.

#### Cell lines

The human monocytic leukemia THP-1 cell line can be differentiated into macrophages and has been widely used in immunological studies of monocyte and macrophage functions (Shah et al., 2022). THP-1s are easy to culture and economic to maintain. Assays are readily scalable and allow for large-scale drug screening on intracellular bacteria (Rankine-Wilson et al., 2022). Yet, THP-1s do not account for human genetic diversity and as a cancerous cell-line have limited physiological relevance.

#### Primary cells

Cellular TB immunology research is very often conducted on human macrophages derived from blood monocytes (MDM), whose polarization and ultimate phenotypes upon *Mtb* infection vary with specific culture conditions (Vogt and Nathan, 2011; Murray, 2017). *Mtb* infection of MDMs has elucidated distinct immune escape and protection mechanisms, such as inhibition of phagosome-lysosome fusion (Armstrong and Hart, 1971), phagosome escape (Van Der Wel et al., 2007) and autophagy (Gutierrez et al., 2004). However, since *Mtb* transmits via aerosols, the first cells to be infected *in vivo*, are alveolar macrophages (AMs), which have distinct characteristics and responses to *Mtb* infection than MDMs (Campo et al., 2024; Tomlinson et al., 2012; Papp et al., 2018). AMs can be isolated from bronchioalveolar lavage (BAL) specimens (Dodd et al., 2016); albeit, in limited numbers. Hence, differentiation of blood monocytes into alveolar-like macrophages facilitates ample study of human alveolar macrophage functions (Pahari et al., 2023, 2024). Another macrophage phenotype commonly observed in TB granulomas consists of foamy macrophages (FMs), which result through lipid droplets accumulation. FMs provide a niche for *Mtb* dormancy and persistence and can be differentiated from monocytes in response to mycolic acids (Peyron et al., 2008) or hypoxia (Daniel et al., 2011). Their study has provided insights into the interconnected macrophage and *Mtb* lipid metabolism associated with *Mtb* dormancy and TB reactivation (Daniel et al., 2011; Santucci et al., 2016).

#### Stem cells

The discovery of inducible-pluripotent stem cells (iPSC) continues to revolutionize medical research (Shi et al., 2017; Cerneckis et al., 2024). iPSCs are expandable, genetically editable and can be differentiated into most organ-specific cell types, all while retaining the genetic background of the original donor (Shi et al., 2017). Gutierrez’ lab used CRISPR-Cas9 edited iPSC-derived macrophages to identify a role for *ATG14* (a gene involved in autophagy) as a regulator of phagosome-lysosome fusion (Aylan et al., 2023). This study demonstrates the utility of iPSCs to study *Mtb*-macrophage interaction at single-gene level, which was previously not possible in primary human cells. In combination with *Mtb* reporter strains (detailed below), iPSC-derived macrophages allow for high-throughput assessment of such host-pathogen interactions, as well as assessment of drug penetration, toxicity and efficiency. Furthermore, iPSC can be derived from patients, opening avenues for personalized medicine and cell therapy (Shi et al., 2017; Cerneckis et al., 2024). Finally, iPSC (and other stem cells) make the generation of (patient-specific) organoids possible. The implications of organoids in disease research are discussed below.

### Dual-culture systems

Modeling the first cellular interaction with *Mtb* in the alveolar space seems of particular relevance to elucidate how an infection can be favored or cleared prior to the onset of an adaptive immune response. A system modelling the alveolar barrier was first developed in 1999 (Birkness et al., 1999). Human lung epithelial type II pneumocytes were cultured with endothelial cells in a two-chamber transwell system. The addition of macrophages allowed the characterization of multiple scenarios of *Mtb* translocation across the cellular bilayer (Bermudez et al., 2002). Interestingly, a similar model was transposed for *M. bovis* infections (Lee et al., 2020; Lee and Chambers, 2019). More advanced systems modeling the alveolar space are discussed in the ‘complex models’ section below. In addition, T cell/macrophage dual-culture systems were notably used to highlight the poor recognition of M2-like infected MDMs by *Mtb*-specific autologous T cells (Gail et al., 2023) or the antimicrobial activity of granulysin delivered upon degranulation of γδ T cell clones onto *Mtb*-infected macrophages (Stenger et al., 1998).

### Multi-cellular systems

#### Primary cell profiling

Direct assessment of primary cells (mostly of blood origin) has been leveraged to improve TB diagnostics, assess the impact of co-morbidities and -infections (HIV and diabetes cohorts, helminth co-infections) or to identify immune correlates of protection in vaccine trials. These studies analyzed whole blood, peripheral blood mononuclear cell (PBMC), as well as BAL specimens *in vitro* directly (e.g. by single-cell RNA sequencing) or following infection or stimulation with relevant antigens (Scriba et al., 2017; Walzl et al., 2011). In a pillar study using whole-blood transcriptome analysis, an interferon-driven neutrophil signature associated with active TB disease (Berry et al., 2010). Issued from relevant clinical cohorts, such samples provided valuable insights for disease prediction. However, the potential for mechanistic investigation remains limited, unless combined with *in vitro* models detailed below.

#### *In vitro* and *ex vivo* granulomas

Granulomas constitute the hallmark of human TB immune reactions. These aggregates of immune and non-immune cells form to physically contain *Mtb*, which may be cleared resulting in a fibrotic or calcified lesion. Otherwise, *Mtb* may persist in a non-replicating state or continue to replicate and eventually exploit the expansion of the granulomatous response to leak into the airways and spread (Ehlers and Schaible, 2012). As such, granulomas hold potential to be tuned towards protectiveness by host-directed interventions. The first granuloma-like structures were generated from PPD-coated beads (Puissegur et al., 2004). Since then, several granuloma models have been developed (Kapoor et al., 2013; Guirado et al., 2015; Tezera et al., 2017), and previously reviewed (Elkington et al., 2019). *In vitro* granuloma models have provided insights into the capacity of LTBI over healthy controls to better control *Mtb* growth (Guirado et al., 2015; Crouser et al., 2017), and to promote *Mtb* dormancy or resuscitation upon exposure to TNF-α antagonists (Kapoor et al., 2013; Arbués et al., 2020a; Tezera et al., 2020). 3D granuloma models restore antimicrobial susceptibility to pyrazinamide (Bielecka et al., 2017) and capture the variable impact of *Mtb* genetic diversity (Arbués et al., 2025). 3D models (Kapoor et al., 2013; Arbués et al., 2020b), unlike 2D ones (Guirado et al., 2015), generate a hypoxic environment that specifically induces *Mtb* to exhibit dormant-like features (Arbués et al., 2021). However, matrix and electrospray 3D technologies (Tezera et al., 2017) hamper the high-throughput-capacity of these models. In contrast, 2D granuloma models are easier to use, which makes them more suitable for drug screening-platforms.

#### Spheroid models

A spheroid model leveraged magnetic cell levitation to generated three-dimensional spheroid granulomas from primary human AMs infected with BCG as “innate” spheroids or with autologous T cells to generate “adaptive” spheroids (Kotze et al., 2021). Interestingly – and unlike the granuloma models described above – spheroids form even in absence of infection and the architecture is altered upon infection by containing an AM-rich core and a cuff of T cells. A miniaturized TB spheroid model allows formation of granuloma-like structures without addition of an extracellular matrix from both cell lines (THP-1, Jurkat) and primary cells (PBMCs) (Mukundan et al., 2021a, 2021). This model may include fibroblasts and was used to study disruption of granuloma formation following HIV co-infection.

#### Alveolar interface systems

Characterizing the alveolar microenvironment and the interaction of epithelial cells with *Mtb* and immune cells is crucial to decipher host-pathogen interactions at the onset of infection. An air-liquid interface model of human primary bronchial epithelial cells (ALI-PBEC) was used to compare the infection of epithelial cells across mycobacterial species (Barclay et al., 2023). These ALI-PBECs incorporate a mixture of PBEC cells from various donors to account for human diversity. Interestingly, this model demonstrated that alveolar cells’ secretome attracts neutrophils, highlighting an immune regulatory function in response to infection. Besides, an experimental human lung tissue model of epithelial cells and fibroblasts that produces extra-cellular matrix (ECM) and secretes mucus, was used to study dendritic cell function and monocyte migration, revealing that granuloma formation was dependent on ESAT-6 secretion by mycobacteria (Parasa et al., 2014; Hoang et al., 2012).

### Complex models: from organoids to organs-on-a-chip

While multicellular systems can account for genetic diversity and assess interactions between various immune cells, they do not apprehend the structural features of the lung (Kim et al., 2024). Organoids are functional 3D *in vitro* replicates of human organs that self-organize and self-renew (Clevers, 2016). They constitute very promising platforms that can bridge between animal models and human clinical trials (Thangam et al., 2024), thereby contributing to reducing animal use (3R) and research costs. They can be induced from embryonic/pluripotent (ESC), induced pluripotent (iPSC) or organ-specific adult stem cells (ASC) (Clevers, 2016). For lung organoids, one distinguishes alveolar, airway and whole-lung organoids. The application of these systems covers everything from basic research to regenerative medicine. Importantly, organoids can be generated from healthy and ill tissues to establish differential biomarker expression and further our understanding of disease pathophysiology and cure through drug screening. Regarding the latter, patient-specific ASC-derived organoids are of particular relevance to test personalized interventions. iPSC-derived organoids on the other hand, allow for genetic recombination of progenitor cells prior their differentiation and assessment of the functional contributions of the knock-in or knock-out out genes. In recent years, organoids have gained momentum as tools to model infectious diseases. For TB, a human bronchial airway organoid model was used to demonstrate the increased fitness of *M. abscessus* over *Mtb* to invade the airway microenvironment (Iakobachvili et al., 2022). Besides, human lung organoids (LOs) based on a human pluripotent stem cell line allowed the assessment of long-term replication of *Mtb* within THP-1 cells following LO micro-injection (up to 31 days) and were used to validate the potential of two promising HDT pathways in a knock-down approach (Kim et al., 2024). Albeit based on cell lines, this model holds great potential to study protective responses by incorporating ASC/iPSC as well as autologous primary human macrophages and other immune cells of individuals suffering from or resisting TB.

The most advanced *in vitro* systems are organs-on-a-chip, which are microfluidic devices allowing renewal of “body” fluids that are being widely adopted in different fields of research to allow for mechanistic dissection of human diseases and drug treatment (Ingber, 2022). Lungs-on-a-chip can recapitulate the alveolar-capillary interface, with organ-level functions such as breathing-type movements and inflammatory responses to pathogens (Huh et al., 2010). Until a few years ago, organoids and lungs on-a-chip had not yet been used for TB research (Fonseca et al., 2017). Recently, using a murine-based lung-on-a-chip, Thacker et al. demonstrated the protective role of surfactant, an essential factor that cannot be knocked-out in *in vivo* systems, in the control of *Mtb* replication (Thacker et al., 2020). Their findings shed light on the increased TB susceptibility of elderly and smokers. In combination with a mouse *in vivo* model and agent-based modelling, they further enlightened dynamics of *Mtb* cording (Mishra et al., 2023). The translational potential of human-based lungs-on-a-chip to study human pathophysiology in TB is underlined by its recent development and use to demonstrate the implication of endothelial cells damage in the pathogenesis of coronavirus infection (Thacker et al., 2021).

### Advanced experimental technologies

Several cutting-edge technologies are being progressively implemented into the various experimental systems to mechanistically dissect their outputs (**Figure 2**). Advances in single-cell RNA sequencing for TB research has been reviewed recently (Pan et al., 2023). Moreover, high-dimensional (spatial) mass cytometry and spectral flow-cytometry, as well as high-throughput and live-cell microscopy are being applied to the TB research field (Aylan et al., 2023; Silva-Miranda et al., 2015; McCaffrey et al., 2022; Ogongo et al., 2024). These tools are now commercialized as kits enabling standardized and reproducible read-outs for clinical studies and allowing data concatenation and comparison across sites (e.g. Standard Biotools’ MDIPA, BD Rhapsody Targeted human immune gene panel). The body of cloud-based platforms offered by companies developing these kits is almost systematic while sequencing costs are dropping substantially, rendering these systems more accessible.

Computational tools to analyze high-dimensional data are also growing by the day. These tools are openly shared on web-based platforms (GitHub, Bioconductor, …) and generously maintained by their developers, encouraging a fruitful, collaborative environment, promoting advances in scientific research globally. Many come with step-wise guides or examples, making them easy to use, even for non-computational experts.

Remarkably, an array of reporter *Mtb* strains has been constructed and generously shared upon request to track bacteria while reporting on their viability or response to immunological stresses (Abramovitch, 2018; Aylan et al., 2023; Bryson et al., 2019; Sukumar et al., 2014). Fluorescent *Mtb* strains greatly facilitate microscopy-based high-throughput read-outs. Furthermore, barcoded *Mtb* strains were built to demonstrate that single bacteria lead to individual granulomas in NHPs (Martin et al., 2017; Bromley et al., 2024). Like knockout mutants, *Mtb* reporter strains provided substantial insights into the host-pathogen interaction occurring in their respective models, by delivering complex microbiological endpoints associated to immunological responses or environments.

## *In silico* approaches

Beyond wet-lab systems, the computational field has delivered algorithms and deep-learning models that can be trained with experimental and clinical data to infer immune cell interaction and predict drug efficacy (Linderman and Kirschner, 2015). As such, computational models hold great potential to guide research and tailor funding towards the most promising leads (Linderman and Kirschner, 2015). A hybrid multi-scale model of granuloma formation (called *GranSim*) integrated experimental and computational modeling to study cytotoxic and regulatory signaling dynamics in granulomas (Warsinske et al., 2017), as well as to study granuloma-associated fibrosis, predicting a potential role for macrophage-to-myofibroblast transformation (Evans et al., 2021). A further development of this model, *HostSim*, integrates multiple physiological and time scales, tracking cellular, granuloma, organ and whole-host events (Joslyn et al., 2022). *HostSim* may provide valuable predictions of understudied lymph node granuloma dynamics (Krupinsky et al., 2025) and even allow conducting virtual clinical trials (Michael et al., 2024). *In silico* approaches seem especially promising when integrated with multi-omics read-outs of experimental models to reproduce an observed phenotype (Chen et al., 2023). In an elegant approach, an agent-based model fed with data from advanced *in vitro* systems was used to assess the immunological determinants enabling better control of *Mtb* growth by macrophages in a spheroid granuloma model (Kotze et al., 2021; Petrucciani et al., 2024).

## Discussion

### Further considerations and future perspectives

#### iPSC limitations

The opportunities awarded by the possible use of stem cells to study infectious diseases appear endless. Nevertheless, some limitations may likely arise, as iPSC-derived macrophage function was notably found to differ depending on culture conditions, which ultimately affected *Mtb* growth (Bussi et al., 2024). It is presumable that organoid functions will be affected similarly and preclude usage of unphysiological conditions to increase a translation potential of the generated data *in vivo*.

#### Strain variation

Likewise, and often overlooked, the selection of the infecting strain may be consequential. Clinical and epidemiological data indicate that the strain genetic background may influence the nature and extent of TB disease (Peters et al., 2020). *Mtb* global phylogenetic diversity contrasts the limited diversity of strains used in TB research, which is mostly conducted on a handful of laboratory-adapted *Mtb* strains (e.g. H37Ra, H37Rv, Erdman, CDC1551, HN878) (Gagneux and Small, 2007) or related species (e.g. *M. bovis* BCG, *M. smegmatis*, *M. marinum*, *M. avium*). While this enables comparison of findings across laboratories, *Mtb* strain variation has implications for the efficacy of TB vaccines, diagnostics and host-directed therapeutics (Tientcheu et al., 2017). Clinical *Mtb* strains can be routinely isolated and identified from sputum samples, as well as less invasive tongue swaps or mask samples that are being investigated to improve TB diagnosis (Nogueira et al., 2022). Ideally, this variable will be systematically included while advancing immunological studies into complex cellular systems, as previously appreciated within simpler experimental systems (Portevin et al., 2011; Reiling et al., 2013; Wang et al., 2010; Arbués et al., 2025; Hiza et al., 2024; Romagnoli et al., 2018).

#### Co-prevalent microorganisms

Gut microbiome dysbiosis as well as co-infections, such as helminths, are gaining recognition as potent influencers of immune responses to vaccination and *Mtb* infection (Cadmus et al., 2020; Eribo et al., 2020; Feng et al., 2025). Microbiome interactions may be particularly relevant in further understanding the contribution of innate-like lymphoid (ILC) and mucosa-associated invariant T cells – two cell types tightly linked to microbiota (Mori et al., 2021) –, to TB protective immunity. Therein, microbiota might constitute a point of manipulating mucosal responses towards protectiveness. An in-depth overview of the interactions between microbiomes and tuberculosis has been provided elsewhere (Mori et al., 2021; Namasivayam et al., 2018).

#### Epigenetics

Another emerging concept relates to epigenetic changes resulting from BCG vaccination and chronic infections (such as TB), which respectively increase non-specific protection through innate immune training (Ziogas et al., 2025) or promote post-TB morbidity and mortality (Abhimanyu et al., 2024; Bobak et al., 2022). Recent findings in guinea pigs and humans indicate that *Mtb* infection induces premature epigenetic aging, which could support post-disease morbidity (Bobak et al., 2022). Metabolic responses, e.g. tricarboxylic acid (TCA) cycle (Abhimanyu et al., 2024), are crystallizing as important drivers of these epigenetic changes. Particularly in the elderly, epigenetic remodeling by host-directed immunomodulation constitutes a promising therapeutic approach (Romero-Rodríguez et al., 2025). Interestingly, epigenetic changes have been associated with resisters' monocytes in independent studies (Zhang et al., 2021; Seshadri et al., 2017). TB research should further investigate epigenetic changes occurring following *Mtb* infection, the effect of these changes on subsequent immune responses and their potential suitability for therapeutic intervention.

### Concluding remarks

TB immunological research has to distinguish between protective immunity in terms of (i) natural protection against primary infection, (ii) vaccine-induced protection against infection or disease, (iii) natural or vaccine-induced protection against disease progression. How different samples and models may facilitate the identification of correlates of protection for vaccine development has been recently reviewed by Wang et al., 2024. Advanced *in vitro* approaches were not featured, and we aimed to cover this gap in the present review.

**Figure 3:**
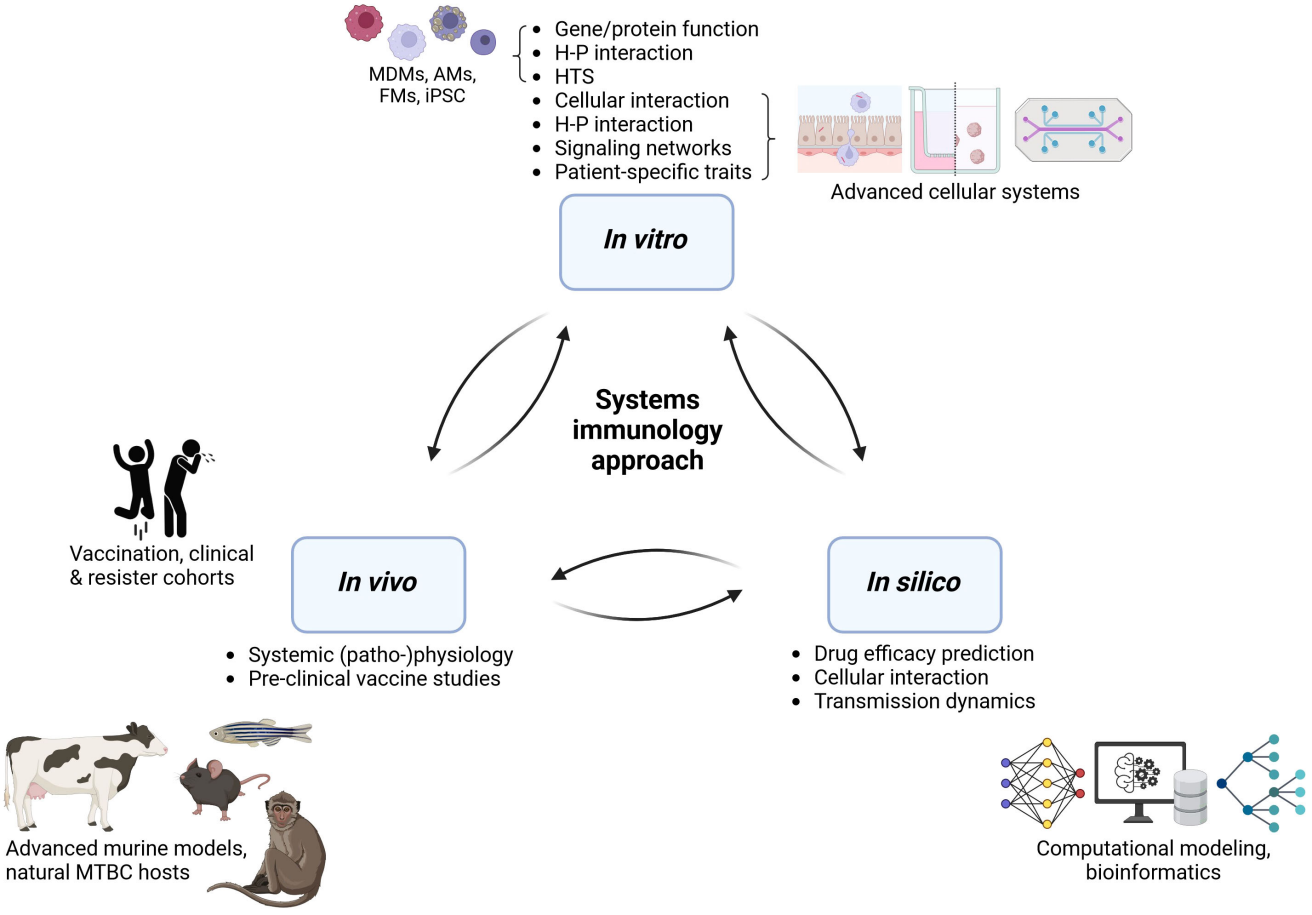
An integrative approach to decipher human protective immunity in TB. H-P, host-pathogen; HTS, high-throughput screening; MDMs, monocyte-derived macrophages; AM, alveolar macrophages; FM, foamy macrophages; iPSC, induced pluripotent stem cells; MTBC, *Mycobacterium tuberculosis complex*. Generated with biorender.com.

Individual models of TB immunology cannot capture all facets of human TB pathology. However, they allow us to perform mechanistic investigations of specific factors and to evaluate the impact of human diversity. An ultimate model would likely integrate multiple approaches, shining light on protective immune traits from all necessary angles (**Figure 3**). We posit that an iterative systems approach is necessary to dissect the many facets of human protective immunity in TB, where *in vitro*, *in vivo*, and *in silico* approaches are gradually being integrated. Collective efforts should extract the most of human *in vitro* studies by sharing and making use of clinical samples and combining the generated data with fast-growing computational modeling field in order to: (1) Extract protective traits from human-based *in vitro* systems, aided by computational modeling. (2) Perform mechanistic investigations in advanced multi-cellular organoid systems that may feed predictive *in silico* models to capture the spectrum of human outcomes in TB. (3) Translate and confirm findings *in vivo* in animal models prior scale-up for clinical safety and efficacy evaluation in human trials. Such an integrative, collaborative approach may pave the way for innovative interventions needed to reduce TB burden globally.
